# Pathology and Treatment of Ringworm

**Published:** 1888-12

**Authors:** 


*Pathology and Treatment of Ringworm.* By GEORGE THIN, M.D. London: J. & A. CHURCHILL. 1887.

Dr. Thin's skill in microscopical investigation, and in the delineation of the results thereof, are well displayed in this monograph on ringworm. In simple language he gives a clear description of the different varieties under which this disease is met with in this and other countries, both in man and other domesticated animals whose skins are capable of forming a nidus for the parasite. Upon the subject of treatment Dr. Thin counsels above all things mild measures and patience; energetic plans of cure he does not think the severity of the disease is ever great enough to warrant. Even Dr. A. J. Harrison's method he thinks unnecessarily strenuous, and occasionally followed by untoward results. Epilation he does not consider to be of any use. In treating of the different substances and preparations which he has employed as applications, he leaves his readers at times in some uncertainty as to his exact meaning; thus, concerning carbolic glycerine: "the strength was 7 drams (of carbolic acid) to the ounce. I have used in some instances carbolic glycerine in considerably greater strength than even in this case," &c. It is difficult to see how a *considerably greater* strength than 7 drams of carbolic acid to the ounce could be obtained. It would have been better if such ambiguity had been avoided by all formulæ having been given in regular prescription form. With the exception of this one slight defect, we can thoroughly commend the book.

